# Evaluation of pain during gingival depigmentation using diode laser and conventional scalpel technique

**DOI:** 10.6026/973206300213264

**Published:** 2025-09-30

**Authors:** Rafat Sultana, Anindita Banerjee, Rajat Sehgal, Prabhat Kumar Singh, Toshi, Shakeb Khan Afridi

**Affiliations:** 1 Department of Dentistry, Anugrah Narayan Magadh Medical College and Hospital, Gaya, Bihar, India; 2 Department of Periodontics, Buddha Institute of Dental Sciences and Hospital (BIDSH), Patna, Bihar, India; 3 Department of Periodontics, Sehgals Dental Clinic, Delhi, India; 4 Department of Oral Pathology, Hazaribagh College of Dental Sciences and Hospital, Hazaribagh, Jharkhand, India

**Keywords:** Gingival depigmentation, VAS, laser

## Abstract

The pain score during gingival depigmentation by diode laser and scalpel is of interest. Hence, the total numbers of patients included were 20. The quadrant undergoing depigmentation via diode laser and scalpel was designated as Group-I and Group II respectively. Data shows that both the conventional scalpel technique and the diode laser achieved similar result in terms of pain perception.

## Background:

Smile is an expression denoting pleasure, sociability, happiness or amusement and can reflect self-confidence of an individual. Smile is not only determined by the shape, color, shade and position of the teeth in the dental arch but also by the way how gingival tissue appears [1]. A dark or black colored gum is one of the concerns of patients reporting to dental clinics [2]. The normal physiologic color of healthy gingiva has been described as coral pink, salmon pink with physiologic variations of melanin pigmentation [3]. Gingival hyperpigmentation is also termed as physiological or racial pigmentation, it occurs due to excessive accumulation of melanin in the basal and supra-basal layers of the epithelium. Gingival pigmentation occurs as a diffuse, deep-purplish discoloration or as irregularly shaped brown and light brown patches. It may appear in the gingiva as early as 3 hrs after birth and often is the only evidence of pigmentation [4]. The prevalence of melanin pigmentation varies between 0% to 89% in different populations with regard to ethnic factors and tobacco usage [4]. Gingival depigmentation is a periodontal plastic procedure whereby the gingival hyperpigmentation is removed or reduced by various techniques such as De-epithelization (Scalpel technique, Gingival abrasion technique using diamond abrasive point, Combination of the scalpel and bur), Gingivectomy, Gingivectomy with free gingival autografting, Acellular dermal matrix allograft, Electrosurgery, Cryosurgery, Chemical agents, Laser (Nd:YAG, Semiconductor diode laser, CO2 laser, Argon laser) etc. One of the first and still popular techniques to be employed in the surgical removal of undesirable pigmentation using scalpels. The procedure essentially involves surgical removal of gingival epithelium along with the layer of underlying connective tissue and allowing the denuded connective tissue to heal by secondary intention. The new epithelium that forms is devoid of melanin pigmentation. The term LASER is an acronym for "Light Amplification by Stimulated Emission of Radiation". Laser is a device that emits light through the process of optical amplification based on stimulated emission of photons. There are several types of LASER available. The diode laser does not interact with dental hard tissue, making it an excellent soft tissue surgical laser indicated for cutting and coagulating gingiva and oral mucosa and for soft tissue curettage or circular debridement. The advantage of diode laser over the other laser is the smaller size of the units as well as the lower financial cost [5]. Patient discomfort and post-operative pain is a common clinical event associated with many periodontal procedures. Therefore, it is of interest to conduct a research in which the two most commonly used methods of gingival depigmentation i.e. the scalpel technique and diode laser are compared on the Visual Analogue Scale (VAS) for pain assessment.

## Materials and Methods:

A split mouth randomized clinical study, was carried out in the Department of Periodontology, Buddha Institute of Dental Sciences and Hospital, Bihar involving 20 subjects, out of which 8were females and rest were males. A prior clearance from the institutional ethical committee was obtained. Twenty subjects complaining of unaesthetic dark gums on the basis of the following inclusion and exclusion criteria were selected for the study. Gingival depigmentation procedure was planned in labial gingiva of maxillary and mandibular sexant.

## Inclusion criteria:

[1] Subjects complaining of hampered esthetics due to "black gums".

[2] Patients with good oral hygiene.

[3] Subjects having healthy periodontium.

## Exclusion criteria:

[1] Pregnant and lactating women.

[2] Medically compromised subjects.

[3] Subjects who consumed tobacco in any form.

[4] Subjects having history of postsurgical keloid.

[5] Patients in whom, gingival hyperpigmentation is associated with other syndromes, lesions and conditions.

## Methodology:

Before depigmentation procedure, a detailed periodontal case-history was taken. Oral prophylaxis was performed and oral hygiene instructions were given to all the patients. All patients were recalled after one week. Dummett-Gupta oral pigmentation index (DOPI) was used to grade the level of gingival hyperpigmentation [6].

## Scoring criteria for DOPI:

[1] No clinical pigmentation.

[2] Mild clinical pigmentation.

[3] Moderate clinical pigmentation.

[4] Heavy clinical pigmentation.

Pigmented segment on labial side of maxillary and mandibular arch was divided into two halves. The divided sites were randomly allotted for depigmentation procedure either with diode laser or scalpel technique by flips of a coin. The sites undergoing depigmentation via diode laser was designated as Group-I, whereas the sites where scalpel technique was used was designated as Group-II.

## Clinical procedures:

The area was anesthetized with 2% lignocaine hydrochloride. After adequate anesthesia was achieved, depigmentation procedure was performed in the allotted sites with either of the technique or instrument used.

## Diode laser technique:

The depigmentation was carried out with diode laser with 980 nm wavelength (CHEESE DEN10B®). The laser tip was used in contact mode on the pigmented part of gingiva. The ablation was performed using paint brush strokes in hyper pigmented areas. Sterile gauze soaked in saline was used to remove the char formed over the surface of the surgical area. Thorough examination was carried out with magnifying glass to make sure that all pigmented epithelium was removed. The depigmented area was covered with a periodontal dressing.

## Conventional scalpel technique:

After adequate anaesthesia, with a surgical blade no.15 or 11complete gingival epithelium was removed by slicing the gingiva. The depigmentation was carried out where gingival hyperpigmentation was present. Care was taken to ensure that all the pigmented epithelium was removed. These sites were also exposed to laser in non-contact mode to keep the subjects blind regarding the particular techniques used. Hemorrhage during the surgical procedure was controlled by direct pressure with sterile gauze. The depigmented area was covered with a periodontal dressing (COE-PAK® GC FUZI). For both the groups analgesics were prescribed to control pain if required and patients were asked to follow postoperative instructions. Patients were instructed to avoid eating of hot and spicy foods for the first 24 hours and were advised to use chlorhexidine mouthwash twice daily. All patients were recalled after 24 hrs and 7 days for visual analogue scale (VAS) score. Both procedures were done on the same day and patients were not aware regarding which procedures were used at which site.

## Method of scoring:

The pain experienced during the procedure was recorded at 2 hrs, 24 hrs and 7 days after the surgical procedure by using VAS. Related to the pain scale, the left end point was nominated as "no pain" whereas the right end point was nominated as "unbearable pain," where VAS score: 0 = no pain; 1-3 = slight pain; 3-6 = moderate pain; and 6-10 = severe pain.

## Statistical analysis:

Data was entered in Microsoft excel and Repeated Measures ANOVA with Tukey's Multiple Comparison Test and student t-Test were analyzed by using statistical analysis software Graphpad Prism (Version 5). 'p' value of less than 0.05 was accepted as indicating significance.

## Results:

The mean VAS score in group I at 2 hours, 24 hours and 7 days postoperatively was found to be 0.95, 0.20 and 0.00 respectively. The difference among the mean was found to be statistically significant. The VAS score was compared at different point of time in Group-I. A decrease in mean pain score was observed from 0.95 (at 2 hours) to 0.20 (at 24 hours) which further reduced to zero at 7 days within the Group-I. The difference among the means of VAS score at 2 hours when compared to 24 hours was found to be statistically significant (p<0.05). Similarly, the difference among the mean at 2 hours when compared to 7 days was also found to be statistically significant (p<0.05). Whereas the difference among the mean of VAS score at 24 hours when compared to 7 days was found to be statistically non-significant (p>0.05). The mean VAS score in group II at 2 hours, 24 hours and 7 days postoperatively was found to be 1.75, 0.85 and 0.00 respectively. The VAS score was compared at different point of time in Group-II. A decrease in mean pain score was observed from 1.75 (at 0-2 hours), to 0.85 (at 24 hours) which further reduced to zero at 7 days within Group-II. The difference among the means of VAS score at 2 hours when compared to 24 hours was found to be statistically non-significant (p>0.05). Whereas, the difference among the mean of VAS score at 2 hours when compared to 7 days was found to be statistically significant (p<0.05). But again, at 24 hours when compared to 7 days the difference among the mean of VAS score at was found to be statistically non-significant (p>0.05). Inter-group comparison of vas scores was carried out at the three different designated time interval. AT 2 hours: The mean VAS scores in group I & II at 2hours was found to be 0.95 and 1.75 respectively. The difference among the meanswas found to be statistically non-significant (p>0.05). AT 24 hours: The mean VAS scores in group I & II at 24hours0.20 and 0.85 respectively. The difference among the means was found to be statistically non-significant (p>0.05). AT 7 days: The mean VAS scores in Groups I and II at 7 days was found to be zero.

## Discussion:

The present clinical investigation is a split-mouth randomized clinical comparative study. Twenty patients were surgically treated using scalpel and diode laser. In this study, intra-group comparison of pain level at different point of time i.e. 2 hours, 24 hours and 7 days was done in group-I (Laser). A statistically significant difference was found in the levels of pain at 2 hours, 24 hours and 7 days. These results could not be compared to any literature due to non-availability of any such data in any study sourced by the present author. Even though the above mentioned reduction in pain is logical to presume, as after any surgical procedure a cascade of cellular events are set in motion. These events begin as acute inflammation and cumulate in healing. The signs of inflammation are (pain, heat, redness, swelling) prominent in the beginning and reduce as the tissue recovers and healing takes place. In a study conducted by Suragimath, Lohana and Varma, the efficacy of gingival depigmentation procedure using conventional scalpel technique was compared with Diode laser. A sterile inflammatory reaction was observed in the gingival tissues following use of diode laser. With diode laser haemostasis was achieved easily and there was a relatively clean operating fields as blood vessels in the vicinity of diode laser tip were sealed. The increased pain perception immediately after surgery might be attributed to the fact that laser has the ability to cut and coagulate tissues. After 24 hours there is a decrease in the pain perception due to the protein coagulum formed on the wound surface that act as a biological dressing and seals the ends of the capillaries and venules. A similar intra-group comparison of pain level at different time intervals i.e. 2 hours, 24 hours and 7 days was done in group-II (Scalpel). A statistically non-significant difference was found in the levels of pain at 2 hours, 24 hours and 7 days. Any study discussing the intragroup comparison of pain levels at different time intervals could not be found. More studies are required to elaborate such results. The pain perception at 2 hours after surgery might be due to blood loss and wide open surgical wound. The healing process after surgery by secondary intention is responsible for a decrease in pain level after 24 hours and 7 days. Also, the decrease in pain is again due to progression of cellular events as discussed above [1]. Comparison of the mean VAS score at different point of time (2 hrs, 24 hrs and 7 days) post-operatively in both the groups was found to be non-significant.

In the year 2014, a study conducted by Ribeiro *et al.* concluded that although, Nd:YAG laser or the scalpel technique may be successfully used for the treatment of melanin gingival hyperpigmentation. However, the use of the Nd:YAG laser presented advantages in terms of less discomfort/pain during the posttherapy period and a reduction of treatment chair time [7]. Gul *et al.* in 2019 in a review mentioned that although, the surgical stripping has been the conventional treatment of choice for gingival depigmentation, but review showed that new techniques are equally effective or even better. Laser especially diode laser was the most frequently used technique and showed better esthetic outcomes, less pain, faster healing, and patients' preference and satisfaction after treatment. However, laser showed more regimentation at 6-month evaluation [8]. Alhabashneh *et al.* [9] in a study concluded that both Er:YAG laser and scalpel technique achieved similar outcomes regarding the efficacy of gingival depigmentation, postoperative pain perception, and the time required for the treatment. Laser therapy required more advanced technology and was associated with higher financial costs. Therefore, the scalpel technique was still considered the gold standard treatment for gingival depigmentation.

Biswas, Subhradip 2024 [10]; conducted a study to compare the pain score using the VAS and the recurrence rate of gingival pigmentation, using the three different techniques, i.e., scalpel, laser and electrosurgery. Results revealed the pain intensity was up high in patients treated with scalpel compared to laser and electrocautery, which was statistically highly significant. The findings suggested that laser depigmentation still serves to be the best modality considering the bloodless field and reduced postoperative pain. Furthermore, Devadharshini 2025 [11]; in their study concluded that Diode laser treatment demonstrated superior aesthetic outcomes and reduced postoperative pain compared to the scalpel technique, along with more effective long-term pigmentation controlwhich was not in accordance to that reported in our study wherein both the procedures did not result in any postoperative complication and the gingiva healed uneventfully. The choice of technique to be used mainly depends on the operator's choice as laser technique requires armamentarium and is associated with higher financial cost whereas scalpel technique is more economical. However, a longitudinal multi-centric study with more number of patients is required to strengthen the outcomes obtained by the study.

## Conclusion:

The conventional scalpel technique and the diode laser used for gingival depigmentation achieved similar result in terms of pain perception. Furthermore, both scalpel as well as laser was equally efficient for depigmentation of the gingiva. Both the procedures did not result in any postoperative complication and the gingiva healed uneventfully.
